# Receptor downregulation and desensitization enhance the information processing ability of signalling receptors

**DOI:** 10.1186/1752-0509-1-48

**Published:** 2007-11-09

**Authors:** Harish Shankaran, H Steven Wiley, Haluk Resat

**Affiliations:** 1 Computational Biology and Bioinformatics Group, Pacific Northwest National Laboratory, Richland WA 99352, USA.; 2 Biological Sciences Division, Pacific Northwest National Laboratory, Richland WA 99352, USA.

## Abstract

**Background:**

In addition to initiating signaling events, the activation of cell surface receptors also triggers regulatory processes that restrict the duration of signaling. Acute attenuation of signaling can be accomplished either via ligand-induced internalization of receptors (*endocytic downregulation*) or via ligand-induced *receptor desensitization*. These phenomena have traditionally been viewed in the context of *adaptation *wherein the receptor system enters a refractory state in the presence of sustained ligand stimuli and thereby prevents the cell from over-responding to the ligand. Here we use the epidermal growth factor receptor (EGFR) and G-protein coupled receptors (GPCR) as model systems to respectively examine the effects of downregulation and desensitization on the ability of signaling receptors to decode time-varying ligand stimuli.

**Results:**

Using a mathematical model, we show that downregulation and desensitization mechanisms can lead to tight and efficient input-output coupling thereby ensuring synchronous processing of ligand inputs. Frequency response analysis indicates that upstream elements of the EGFR and GPCR networks behave like low-pass filters with the system being able to faithfully transduce inputs below a critical frequency. Receptor downregulation and desensitization increase the filter bandwidth thereby enabling the receptor systems to decode inputs in a wider frequency range. Further, system-theoretic analysis reveals that the receptor systems are analogous to classical mechanical over-damped systems. This analogy enables us to metaphorically describe downregulation and desensitization as phenomena that make the systems more resilient in responding to ligand perturbations thereby improving the stability of the system resting state.

**Conclusion:**

Our findings suggest that in addition to serving as mechanisms for adaptation, receptor downregulation and desensitization can play a critical role in temporal information processing. Furthermore, engineering metaphors such as the ones described here could prove to be invaluable in understanding the design principles of biological systems.

## Background

Recent systems biology efforts are starting to establish biology as a systems science wherein concepts borrowed from engineering and physical sciences are applied to describe biological systems using an input-output relationship based formalism [1-7]. To contribute to these efforts, in this paper, we use mathematical models for two receptor signaling pathways and show that various aspects of receptor signaling can be investigated using systems science concepts. Using analogies to electrical and mechanical systems, we show that the upstream signal transduction elements for the epidermal growth factor receptor (EGFR) and G-protein coupled receptor (GPCR) systems can be treated as low-pass filter circuits or as mechanical mass-spring-damper systems. As the dynamics of the latter systems have been extensively investigated, establishment of analogies between these biological and physical systems allowed us to develop novel metrics to assess the design principles of receptor signaling systems.

Cells use surface receptor systems to survey their environment. Signal transduction, where information about extracellular stimuli is converted to a biological response, is critically important both in the context of normal cell physiology as well as in pathogenesis. Signaling receptors are known to mediate diverse cellular responses such as migration, proliferation and differentiation. Constitutive and ligand-induced receptor trafficking (*endocytosis*) are important mechanisms for regulating receptor-mediated cell signaling [8-11]. An intriguing property displayed by a number of signaling receptors is that of *ligand-induced endocytic downregulation*, where receptor-ligand complexes are internalized at rates greater than those of free receptors to decrease activated surface receptor levels [12-14]. The EGFR system [15] best exemplifies this phenomenon. Stimulus by extracellular ligand induces a significant increase in EGFR endocytosis [14]. It is intuitively understood that cells use endocytic downregulation as a means to turn the signal off. In support of this notion, there is evidence to suggest that impaired endocytosis and consequent prolonged signaling can have deleterious consequences [16]. However, simply stating that endocytic downregulation is required for turning the signal off may not fully explain the presence of this phenomenon. This is because internalized receptors may continue to signal in the cell interior [17], and it takes time to degrade internalized receptor-ligand complexes. Therefore, cells in fact do not shut the signal off instantaneously by downregulating their receptors. These observations led us to explore whether downregulation can play an additional, hitherto unexplored role in modulating signal transduction.

GPCR *desensitization *is analogous to EGFR downregulation and it has also been proposed to be a feedback mechanism to protect against both acute and chronic receptor over-stimulation [18-20]. The actuating event in GPCR desensitization is the uncoupling of G-proteins from the receptor [19,21]. This step is initiated by the phosphorylation of residues in the cytoplasmic tail of the receptor, and it leads to the abrogation of signaling potential.

In a physiological setting, ligand concentrations may have spatio-temporal variations that could contain information vital to the cell. The ability to accurately process time varying input signals would allow cellular systems to mount appropriate responses to environmental stimuli. In this manuscript we examine the information processing ability of cell surface receptors and restrict our analysis to the early molecular elements in receptor signaling. We make the implicit assumption that the primary task of a receptor is to transduce ligand concentration information without distortion into molecular activation information, which provides the input to downstream signaling processes. The downstream signal transduction events may be complex and could be non-linearly related to, and temporally distinct from the ligand input. For instance, an instantaneous ligand addition could lead to sustained downstream responses due to the non-linearity of the downstream processes. Even under these circumstances, we believe that the temporal distortion is introduced at stages downstream of surface receptor activation and that the cell would still benefit by maintaining a faithful temporal coupling between the input ligand waveform and the upstream molecular signal that it elicits. To summarize, since they constitute the cellular sensory machinery, receptors need to be able to detect the temporal variations in their ligand availability and then convert this information to an input for the further downstream signalling events. The fidelity of this conversion requires that the changes in ligand availability are faithfully reproduced in the input to the downstream stages.

By examining the EGFR and GPCR pathways as model systems, we show that downregulation and desensitization mechanisms can lead to tight and efficient input-output coupling thereby ensuring synchronous information processing. Analysis of the frequency response of the reaction systems indicates that the EGFR and GPCR models behave like low-pass filters that can process inputs below a critical frequency. EGFR downregulation and GPCR desensitization have a qualitatively similar effect on information processing in that they enable the system to accurately process higher frequency inputs. Examination of the governing equations also reveals that the signaling networks are analogous to a classical mechanical system involving the motion of a mass connected to a spring and a damper. In the context of this analogy, downregulation and desensitization can be metaphorically described as phenomena that make the systems more resilient in responding to external perturbations thus improving the stability of the system's resting state.

Our choice of the EGFR and GPCR receptor systems was motivated by the fact that there is sufficient experimental data to model these systems [22-24]. Furthermore, the EGFR and GPCR systems are sufficiently different from each other so as to allow the elucidation of general signal transduction control strategies based on their comparative analysis. Our analysis of these receptor systems suggests that negative regulation mechanisms which have been traditionally viewed in the context of adaptation may in fact play critical roles in temporal information processing. The recognition of the importance of temporal information processing as a design constraint may enable us to better understand the underlying design principles of biomolecular networks. It may be possible to analyze other elements of the signal transduction machinery in the context of information processing using the methodology described here.

## Results and discussion

### Mathematical models for the EGFR and GPCR systems

**Figure 1:**
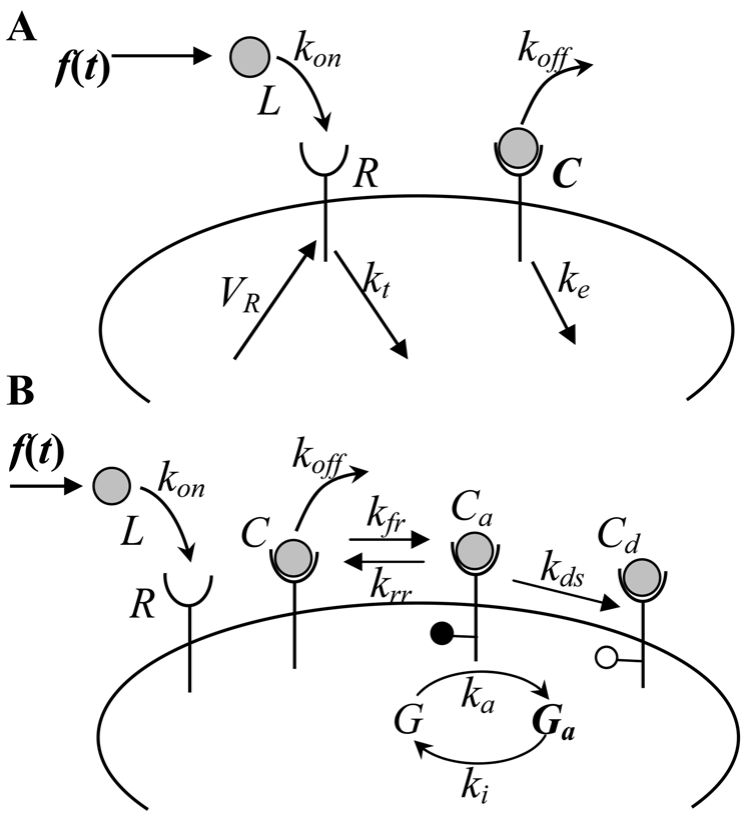
**EGFR and GPCR models**. Schematic descriptions of the EGFR (A) and GPCR (B) systems (see text for details).

Figure 1 provides schematic descriptions of the mathematical models for the EGFR and GPCR systems simulated here. Detailed descriptions of these models and their solution methodology can be found in the Methods section and in the Additional file 1. A brief description of the important features of these models follows.

#### EGFR model

The model for the EGFR (Fig. 1A) includes the reversible binding of receptors *R *to ligand *L *to yield receptor-ligand complexes *C *with forward rate *k*_*on *_and reverse rate *k*_*off*_. Free receptors and receptor-ligand complexes are internalized with rates *k*_*t *_and *k*_*e*_, respectively. Newly synthesized receptors enter the plasma membrane at rate *V*_*R*_. The input *f*(*t*) is the time-dependent ligand entry rate into the extracellular volume *V*, and the number of receptor-ligand complexes at the cell surface *C*(*t*) is the output of the system. In all we have six independent parameters in the model: *k*_*on*_, *k*_*off*_, *k*_*e*_, *k*_*t*_, *V*, and *R*_*T*_. The receptor synthesis rate *V*_*R *_can be derived based on the steady-state condition in the absence of ligand and can be expressed as *V*_*R *_= *k*_*t*_*R*_*T*_. The parameter values employed in our simulations are as follows: *k*_*on *_= 0.097/nM/min, *k*_*off *_= 0.24/min, *k*_*e *_= 0.15/min, *k*_*t *_= 0.02/min, *V *= 4 × 10^-10 ^lt/cell, and *R*_*T *_= 200,000 (see Additional file 1 for details). For the results presented in this paper, all of the parameters are kept fixed at the specified values, and *k*_*e *_alone is varied to examine the effect of downregulation on the system response.

The signal transduction network downstream of the EGFR is quite complex and involves multiple signaling pathways [15,25]. Using *C*(*t*) as a system output parameter is reasonable for a variety of reasons. Firstly, in the case of the EGFR and other signaling receptors, the biological response when a fixed amount of ligand is added has been shown to be proportional to the steady-state level of receptor-ligand complexes at the cell surface [26,27]. Thus, when time-varying ligand inputs are employed it is likely that the dynamic changes in the number of occupied receptors *C*(*t*) (or derivatives thereof) would determine the eventual biological response. Further, in most cases, there are "spare receptors" relative to the number required to produce a maximal biological response, suggesting that the number of occupied receptors at the cell surface will be the controlling factor to downstream events [28]. The specific aspects of *C*(*t*) (steady-state vs. transient; integral of *C*(*t*); etc.), which drive the biological response can be system dependent and would derive from the characteristics of the particular signaling pathway under consideration. Irrespective of the exact link between *C*(*t*) and the biological response, we suggest that an accurate match between the ligand input *f*(*t*) and the upstream system readout *C*(*t*) would be beneficial to the system in the context of information processing.

Here, we use the specific example of the EGFR system to assess the role of receptor downregulation on system response. The effect of varying a single system parameter, the extent of downregulation *D *(= *k*_*e*_/*k*_*t*_) on the EGFR response is examined using the mathematical model shown in Fig. 1A. We note that this model can be applied to other signaling receptors as well, and can also be analyzed to identify the effect of the other system parameters on the response. We have recently performed a comprehensive analysis of this mathematical model to address these issues [29]. Based on our analysis of the governing equations for the model we find that the response dynamics depends upon two fundamental dimensionless parameters *β *= *k*_*e*_*/k*_*off *_and *γ *= *K*_*a*_*R*_*T*_/(*N*_*av*_*V*) where *K*_*a *_= *k*_*on*_/*k*_*off *_is the receptor-ligand affinity (see Eq. 1 in the Methods section for the EGFR transfer function). *γ *is an *avidity parameter *that quantifies the ability of a receptor system to capture extracellular ligand and *β *is a *consumption parameter *that quantifies the ability to consume bound ligand molecules. In this manuscript we analyze the effect of downregulation on the EGFR system by altering *k*_*e*_, the internalization rate of receptor-ligand complexes, which amounts to changing the *β *value while holding *γ *constant. The effect that this change would have on the information processing ability of a specific signaling receptor would depend upon the location of the particular receptor in *β*-*γ *parameter space. We have previously defined the regions of the parameter space where altering *β *would have a significant effect on the response dynamics [29]. Our current results on the effect of downregulation on system response would be applicable to any receptor system in the *β*-sensitive regions of the parameter space (see Figs. 5 and 6 in [29]). We note that the results presented in our earlier manuscript can also be used to assess the effect of altering receptor-ligand binding kinetics and receptor synthesis rates on the response dynamics. This can be accomplished by first identifying the fundamental dimensionless parameter (*β *or *γ *or both) that is altered by changing a specific rate constant and subsequently using Figs. 5 and 6 of our earlier manuscript to quantify the effect of this change.

#### GPCR model

Our GPCR model (Fig. 1B) is adapted from Riccobene et al.'s model for the formyl-peptide (formyl-Met-Leu-Phe or fMLP) receptor system [23]. The model includes the binding of free receptors *R *to ligand *L *with forward rate *k*_*on *_and reverse-rate *k*_*off *_to yield inactive receptor-ligand complexes, *C*. Receptor-ligand complexes are reversibly activated with forward rate *k*_*fr *_and reverse-rate *k*_*rr *_to yield active complexes *C*_*a*_. Activated complexes are irreversibly desensitized at rate *k*_*ds*_. Activated receptor-ligand complexes are also capable of converting inactive G-protein molecules *G *to the active form *G*_*a *_with a second-order forward rate constant *k*_*a *_and reverse rate *k*_*i*_. The parameter values employed in our simulations are as follows: *k*_*on *_= 8.4 × 10^7 ^/M/s, *k*_*off *_= 0.37/s, *k*_*fr *_= 10/s, *k*_*rr *_= 10/s, *k*_*ds *_= 0.065/s, *k*_*a *_= 10^-7^/#/s, *k*_*i *_= 0.2/s, *V *= 4 × 10^-10 ^lt/cell, *R*_*T *_= 55000, and *G*_*T *_= 100000 (see Additional file 1 for details). For the results presented in this paper, all of the parameters are kept fixed at the specified values, and *k*_*ds *_alone is varied to examine the effect of desensitization on the system response.

It should be noted that when ligand is added, receptors are lost due to desensitization and are not replaced by receptor synthesis or recycling terms in this GPCR model. In this regard, we restrict our system to that described by Riccobene et al., and neglect receptor trafficking and synthesis terms for the sake of simplicity. While receptor depletion would have an effect on the magnitude of the response to large ligand doses, for the ligand concentrations examined in this paper receptor depletion is limited and does not affect the results. The system output in our model is the number of activated G-protein molecules, *G*_*a*_(*t*). We note that our major conclusions would still hold if *C*_*a*_(*t*), the number of active surface complexes, was used as the model output. Inclusion of a signal transduction step in the GPCR model enables us to illustrate our analysis strategy for models whose dimensionality is increased by the addition of downstream signaling reactions.

Even though these models are simple, they capture the essential features of the upstream events in the EGFR and GPCR systems. For example, the spatio-temporal distribution of activated receptors obtained using the presented simple EGFR model is very similar to results (not shown) obtained using extended models that we have previously employed [22,30]. We also note that the presented models capture the basic features of the various receptor systems that undergo ligand-induced downregulation and desensitization. Hence, we believe that our conclusions are broadly applicable to other signaling receptors that are subject to negative regulation.

### Receptor downregulation/desensitization increases response speed and improves frequency processing

We hypothesized that signaling receptors are designed to allow cells to generate biological outputs that are tightly coupled to the ligand inputs for a wide range of input patterns. Here, we show that endocytic downregulation and desensitization play a critical role towards this end. They endow the EGFR and GPCR systems with an ability to accurately transduce time-varying ligand doses.

#### Response to ligand impulses

**Figure 2:**
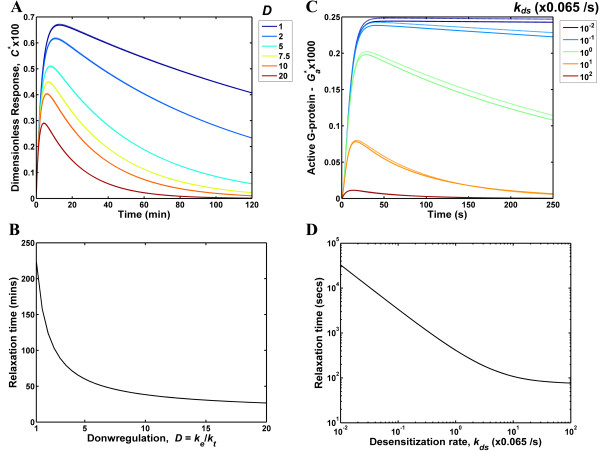
**Impulse response of the EGFR and GPCR systems**. (A&C) The dimensionless response to a ligand impulse was computed for EGFR (A) and GPCR (C). The response is to a ligand impulse of magnitude 0.01*K*_*D *_nM. System responses were computed either by numerically integrating the ODEs governing the system (solid lines) or by inverting the transfer function, *G*(s) of the linearized system of equations (dotted lines). (B&D) The relaxation time is plotted as function of the extent of downregulation, *D *(= *k*_*e*_/*k*_*t*_) for the EGFR (B) and the desensitization rate, *k*_*ds *_for the GPCR (D). Note that the relaxation time in panel D is plotted on a logarithmic scale to capture the large change in GPCR relaxation time magnitude. This plot would be qualitatively similar to panel B when a linear scale is used.

Under *in vivo *conditions cells are likely to be exposed to small, time-varying ligand doses which can be pulsatile and noisy. Such changes in the ligand concentration can be approximated using a series of impulses in the ligand entry rate. In a physiological context, an impulse would be realized when a target cell is exposed to an instantaneous burst of ligand secreted by a neighbouring cell or the cell itself. We assessed the response of the EGFR and GPCR systems to a ligand impulse as a function of the extent of endocytic downregulation and desensitization (Fig. 2). As input, we used an impulse that delivered a total ligand concentration equal to 0.01*K*_*D*_, where *K*_*D *_is the dissociation constant. In other words, the ligand entry rate *f*(*t*) was modelled as a Dirac delta function where the area under the curve is equal to the added ligand concentration. This was implemented by setting the initial ligand concentration *L*(*t *= 0) to equal 0.01*K*_*D*_. This is a small perturbation representative of physiological ligand stimuli. In our simulations we use an extracellular volume of 4 × 10^-10 ^lt/cell. For the EGFR and GPCR receptor systems this ligand concentration and extracellular volume translate to a one-time addition of 5961 and 10612 ligand molecules respectively.

Figure 2A reports the dimensionless number of receptor-ligand complexes C* for the EGFR system for a range of downregulation magnitudes *D *that span the experimentally observed range for this parameter. The *D *value is defined as *D *= *k*_*e*_/*k*_*t *_where *k*_*e *_and *k*_*t *_are the internalization rates of receptor-ligand complexes and free receptors, respectively. For our simulations *k*_*t *_was kept fixed while *k*_*e *_was varied to obtain the desired range of *D *values. We note that our experimental measurements yield *D *values of ~8–10 for cultured epithelial cells [14]. As seen in Fig. 2A, the impulse causes a gradual increase in the system output to a maximum value followed by an exponential decay. Increasing the extent of downregulation results in a response that is faster and has a smaller amplitude while the peak position is rather insensitive to *D*. It should be noted that we use the term "response" to denote the entire time-course of the system output, which includes both the activation and the decay phases. The decrease in amplitude with *D *is caused by the net loss of surface receptors that occurs when ligand-induced endocytosis is higher. We characterize the *response speed *by quantifying the *relaxation time*, which is defined as the time taken for the response to decay to a value 1/*e *of the maximum. The relaxation time for the EGFR system decreases from a value of 224 min to 27 min when *D *is increased from 1 to 20 (Fig. 2B). Thus, downregulation improves the response speed of the EGFR signaling and contributes to better information processing and faster adaptation.

In our simulations of the GPCR system (Figs. 2C and 2D) we varied the receptor desensitization rate *k*_*ds *_in a four-orders of magnitude logarithmic scale to account for the range employed in [23]. These authors suggest a *k*_*ds *_value of 0.065 s^-1 ^as the likely desensitization rate for the fMLP receptor system. Our results for the GPCR model were qualitatively similar to the ones for the EGFR system. Increasing the desensitization rate *k*_*ds *_yielded greater response speeds (Fig. 2D). The relaxation time for the GPCR model varied from 3.3 × 10^4 ^s at *k*_*ds *_= 6.5 × 10^-4 ^s^-1 ^to 77 s at *k*_*ds *_= 6.5 s^-1^.

A faster response would allow the cellular system to accurately decode and transduce inputs consisting of frequent pulses. As an illustrative example, consider a scenario where the EGFR system is exposed to successive spikes in ligand entry spaced an hour apart. With a relaxation time of 27 min (as in the case with *D *= 20), the response to an input pulse would decay completely prior to the arrival of the next spike and this system would generate an output that is a faithful reconstruction of the input pulse train. On the contrary, the system without downregulation (*D *= 1) has a relaxation time of 224 min. So the system would still be responding to the initial stimulus when the second pulse arrives and the overlap between the responses would confound information processing. Based on these observations, we conclude that by increasing the response speed of the respective receptor systems, downregulation and desensitization help to improve information processing fidelity.

Our results also indicate that there is a trade-off between amplitude and response speed. Downregulation and desensitization increase the response speed at the cost of response amplitude. The consequences of this trade-off on the phenotypic response of the cell would require a detailed examination of the link between receptor occupancy and cell phenotype. In the case of the EGFR we have previously shown that the occupancy of only a few receptors is sufficient to trigger mitogenesis [27]. Further, we have shown that in the case of EGFR autocrine signaling, the autocrine ligand release rates are such that the concentration of extracellular ligand and the number of occupied receptors would be low [31,32]. These observations suggest that the EGFR system is capable of generating biological responses even to small amplitude variations in surface receptor occupancy. Thus, we expect that the potential decrease in the signal to noise ratio at large downregulation values will not have a significant impact on the proper functioning of the EGFR system.

As seen in Figs. 2A and 2C, analytical solutions (discussed in the Methods section) of the linearized EGFR and GPCR models (dotted lines) provide reasonable approximations to the numerical solutions (solid lines). Analytical solutions provide us with the advantage of using linear systems theory to understand the design principles of the receptor systems. In the subsequent sections we further characterize the receptor systems using the transfer functions for the linearized versions of these models (see Methods sections for details). We note that we have also computed the response of the EGFR and GPCR systems to higher ligand concentrations (in the order of *K*_*D*_). The analytical approximation is not valid for these concentrations, and this regime was investigated by numerically integrating the governing equations. We found that at high input ligand dosages the parameter dependence of the system response (results not shown) is qualitatively similar to that reported in Fig. 2. Thus, our conclusions would still hold true even at the higher ligand concentrations.

#### Frequency response of the receptor systems

The frequency response of a system defines how the system handles temporal information and is computed by assessing the response of the system to sinusoidal inputs. It should be noted that it is unlikely that the receptor systems studied here would be subjected to purely sinusoidal inputs *in vivo*. However, any time-varying input signal can be partitioned into a set of constituent sinusoidal frequencies using the Fourier transform. So, the frequency response results presented here can be used to assess the response of the receptor systems to any general time-varying input signal.

**Figure 3:**
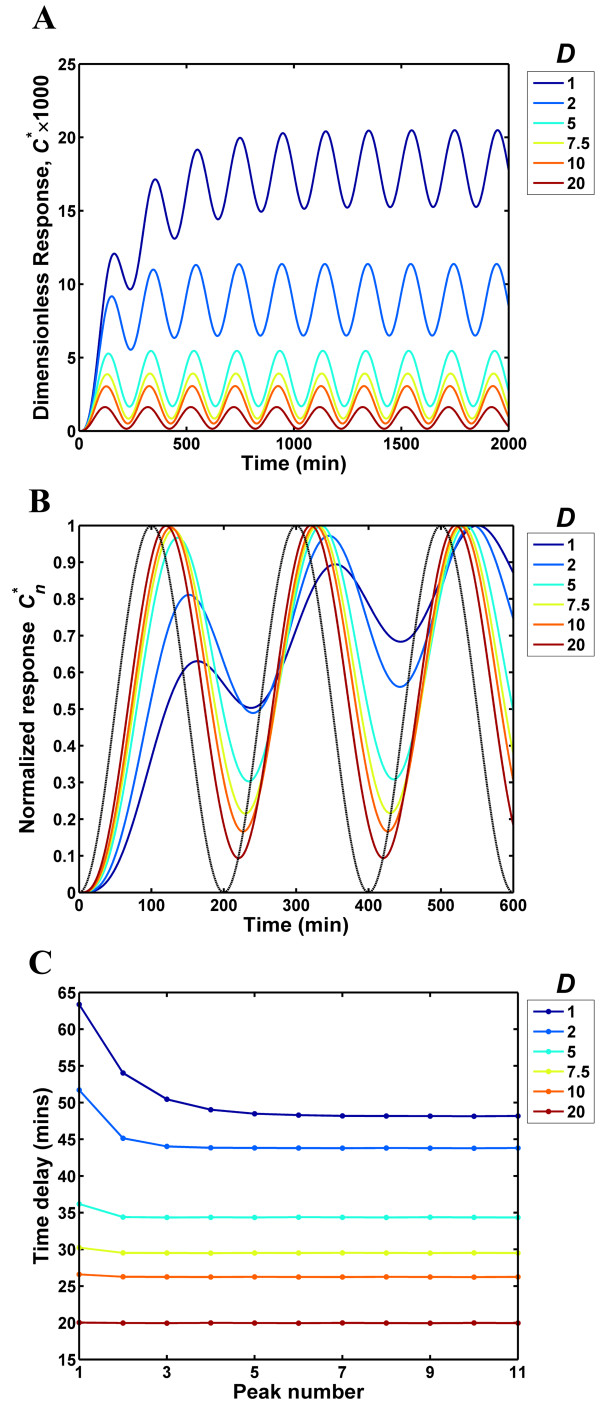
**Characteristics of the EGFR frequency response**. The response of the EGFR system was computed for a sinusoidal input of wavelength 200 min and a dimensionless peak value A* of 0.001. (A) The dimensionless EGFR response is plotted for various downregulation values, *D*. (B) In order to illustrate the time delay between the input and output waveforms, we computed the normalized response *C*_n _* by dividing the dimensionless response C* with the maximum value reached in the chosen time range. This normalized response is plotted as a function of time for the *D *values indicated. The input is denoted by a black dotted line. (C) The time delay between the input and output waveforms was computed using each of the maxima in the response and is plotted as a function of the peak number.

To the best of our knowledge, there are no good estimates of the physiologically relevant input frequencies that the EGFR and GPCR systems are required to process. Here, we use mathematical analysis to identify the range of frequencies that the EGFR and GPCR systems are capable of processing. In order to compute the frequency response, we assessed the effect of endocytic downregulation and desensitization on the system response to non-negative sinusoidal inputs with a peak value *A *nM/min and frequency *ω *= *2*π/*λ *radians/min, i.e., the ligand entry rate was of the form *f*(*t*) = (*A*/2) [1 - cos(*ωt*)] (see Methods section). In dimensionless terms, the input can be written as f*(t*) = (A*/2) [1 - cos(ω* t*)] where A* = *A*/(*K*_*D*_*k*_*off*_), ω* = *ω*/*k*_*off*_, t* = *tk*_*off*_, and *k*_*off *_and *K*_*D *_respectively are the dissociation rate and the dissociation constant for the receptor-ligand binding reaction. Figure 3 highlights the salient features of the frequency response by considering the response of the EGFR system to an input with a dimensionless peak value A* = 0.001 and a wavelength *λ *= 200 min. This input represents a relatively small perturbation which places the model in the linear regime and enables us to employ an analytical solution for the frequency response (Methods section). The reason for our choice of wavelength will be clear when we delve further into the frequency response characteristics of the EGFR system. Briefly, the EGFR system can process this input wavelength faithfully only when the extent of downregulation is large. As seen in Fig. 3A, when confronted with a sinusoidal variation in the ligand entry rate, the EGFR system generates a sinusoidal output waveform. When *D *= 1, the system displays a significant transient and the output rises and eventually settles onto a steady-state oscillation about a constant mean value. Increasing the *D *value shortens the transient response and systems with *D *> 5 display an ability to rapidly settle onto their steady-state. Furthermore, as *D *is increased both the oscillation amplitude and the mean value of the response register a decrease. Results from the first three input pulses in Fig. 3A (*t *= 0 to 600 min) were normalized based on the maximum value reached by the response and plotted in Fig. 3B in order to illustrate the time delay between the input and the output waveforms. For *D *= 1, there is a substantial delay between the input (black dotted line) and the output. The delay decreases when *D *is increased (Fig. 3B). The time delay between the input and output was computed at each of the maxima in the waveforms and is plotted as a function of the peak number in Fig. 3C. Although the time delay is different in the transient and steady-state phases of the system response, the steady-state time delay provides a reasonable estimate of the lag between the input and the output over the time course (Fig. 3C). As the overall response is comprised of transient and steady-state features, we next examine these two aspects individually.

**Figure 4:**
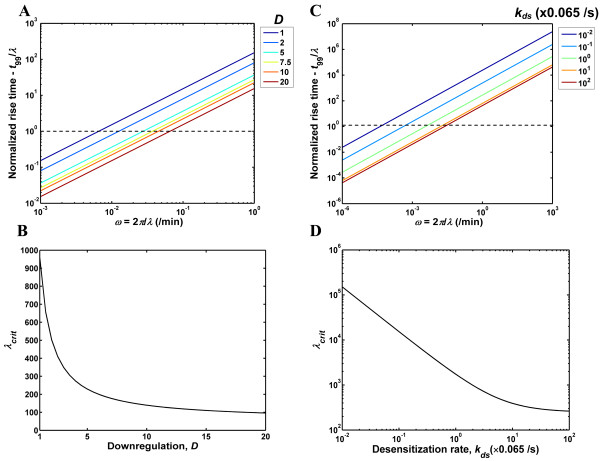
**Transients in the frequency response of the EGFR and GPCR systems**. (A&C) The rise time, *t*_99 _taken for the transient term in frequency response to decay by 99% was normalized with the input wavelength *λ *and is plotted as a function of frequency for the EGFR (A) and GPCR (C) systems. The normalized rise time quantifies the number of input pulses beyond which the steady-state response is reached. The black dotted lines in panels A and C are drawn at a normalized rise time value of 1. (B&D) The input wavelength, *λ*_*crit *_for which the response reaches steady-state in a single input pulse (*t*_99_/*λ *= 1) is plotted as function of the extent of downregulation, *D *for the EGFR (B) and the desensitization rate *k*_*ds *_for the GPCR (D). For input wavelengths *λ *> *λ*_*crit *_the system responds with virtually no transient.

In the transient phase of the frequency response, roughly speaking, the system integrates the input and builds up to its steady-state. Large transient times are detrimental to the fidelity of a receptor system and reflect the system's inability to cope with the variations in the input, i.e. the ligand entry rate in this case. In Fig. 4, we examine the transient phase in the frequency response of the EGFR and GPCR systems. In order to quantify the extent of the transient, we computed the time *t*_99_taken for the transient to decay by 99% from its initial value at *t *= 0 (see Methods section). Thus, *t*_99 _quantifies the time taken for the response to build-up to the steady-state oscillation. Figure 4A reports the normalized rise time, *t*_99_/*λ *as a function of input frequency for the EGFR system. The normalized rise time represents the number of input cycles necessary before the system settles on its steady-state response. As seen in Fig. 4A, for the EGFR system the normalized rise time increases with the input frequency. The normalized rise time and the input frequency display a linear dependence on a log-log plot with a slope of 1 for all the *D *values shown. This is a reflection of the fact the rise time *t*_99 _is nearly independent of the input frequency *ω*. When *D *is increased, the transient shows a marked decrease, with the normalized rise time showing an order of magnitude change between *D *= 1 and *D *= 20 at any given input wavelength. We computed the input wavelength, *λ*_*crit *_for which the response reaches steady-state in a single input pulse, (i.e. *λ *at which *t*_99_/*λ *= 1). For input wavelengths that are greater than *λ*_*crit *_the system will respond with virtually no transient. In Fig. 4B, *λ*_*crit *_is plotted as function of the extent of downregulation *D *for the EGFR system. For *D *= 1, the system can handle inputs with wavelength greater than ~1000 min without a significant transient (Fig. 4B). For *D *> 6, *λ*_*crit *_is less than 200 min with *λ*_*crit *_= 96 min at *D *= 20. In other words, the EGFR system generates a steady-state response with virtually no transient to input pulses of wavelength larger than 200 min when the extent of downregulation is greater than 6. These findings are in line with the results shown in Fig. 3 for the EGFR frequency response. The transient characteristics of the GPCR system (Figs. 4C and 4D) are qualitatively similar to that of the EGFR. When *k*_*ds *_is increased from *k*_*ds *_= 6.5 × 10^-4 ^s^-1 ^to 6.5 s^-1^, the normalized rise time decreases by nearly three orders of magnitude at any given input wavelength (Fig. 4C). The critical wavelength *λ*_*crit *_beyond which the system can process inputs without a significant transient, decreases from 1.51 × 10^5 ^s at *k*_*ds *_= 6.5 × 10^-4 ^s^-1 ^to 263 s at *k*_*ds *_= 6.5 s^-1 ^(Fig. 4D). Overall, increasing the extent of downregulation for the EGFR and the desensitization rate for the GPCR decreases the transient time in the frequency response.

**Figure 5:**
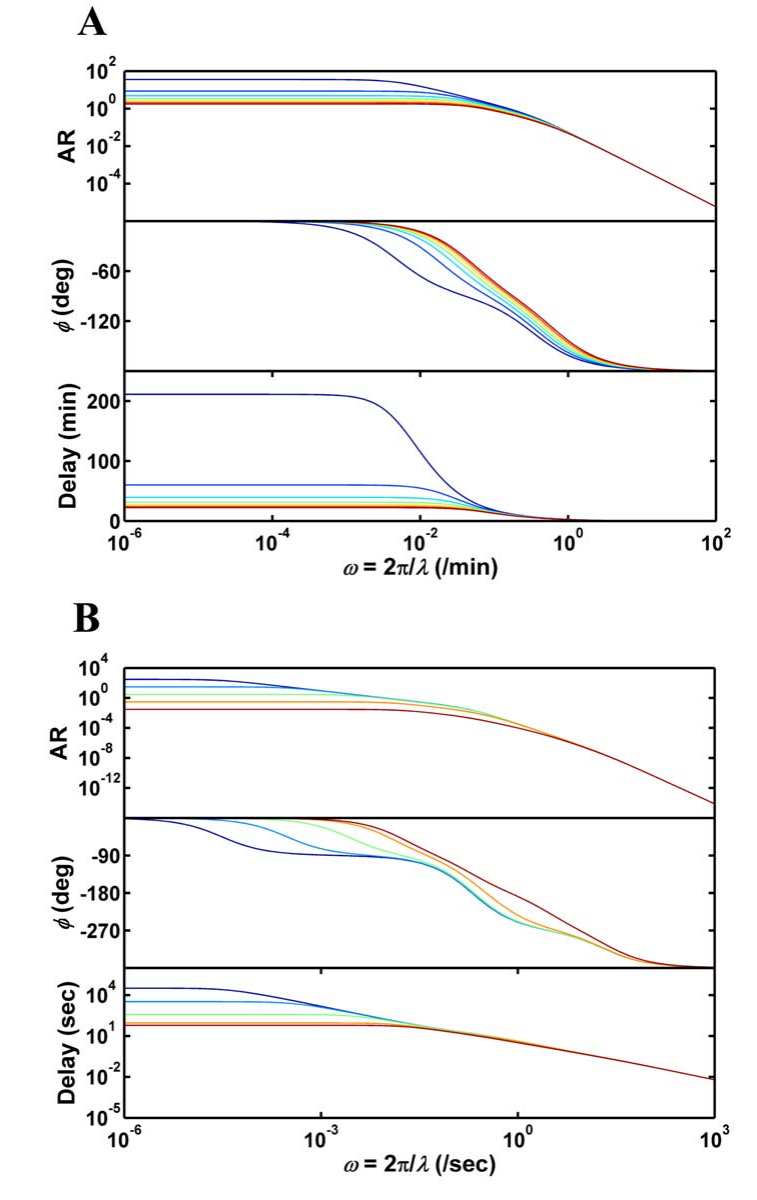
**Steady-state characteristics of the EGFR and GPCR frequency response**. The amplitude ratio *AR*, the phase lag, *ϕ *and the steady-state time delay between the input and output waveforms are plotted for the EGFR (A) and GPCR (B) systems. The *D *and *k*_*ds *_values corresponding to each of the colored lines for the respective receptor systems are the same as the ones indicated in Figs. 2 and 4.

Next, we examined the steady-state characteristics of the frequency response of the EGFR and GPCR systems (Fig. 5). When presented with a sinusoidal input with frequency *ω*, a receptor system with transfer function *G*(*s*) transduces the input to a steady-state output with an amplitude ratio *AR *and a phase lag *ϕ*. Notingthat *AR *and *ϕ *are respectively the magnitude and phase of the complex number *G*(*iω*), we computed these quantities and the steady-state time-delay *t*_*delay *_(= *ϕ*/*ω*) between the input and output waveforms for the EGFR (Fig. 5A) and GPCR (Fig. 5B) systems using their respective transfer functions (Methods section). The frequency response results are plotted as classical Bode plots in the two upper panels of Figs. 5A and 5B. *As seen in these plots, both receptor systems clearly behave as low-pass filters with the frequency processing range increasing with downregulation and desensitization*. The bandwidth frequency *ω*_*BW *_is defined as the frequency at which the output amplitude drops to 70.7% of the zero frequency amplitude (DC gain). For a low pass filter, inputs with frequency lower than *ω*_*BW *_are transduced with a nearly constant amplitude ratio, while inputs with frequency greater than *ω*_*BW *_are significantly attenuated. It should be noted that although the amplitude ratio drops beyond *ω*_*BW *_this does not mean that the system does not generate a significant output. At steady-state the system will display a significantly attenuated oscillation about a non-zero mean value, which is independent of the input frequency but is a function of *D*. Thus, beyond *ω*_*BW *_the system ceases to generate an output that resembles the input waveform and the overall response will simply be a transient rise to a constant mean value. In other words, *ω*_*BW *_quantifies the frequency range where the system responds with high fidelity to the input signal. For the EGFR system, the bandwidth frequency increases from 4.8 × 10^-3 ^min-^1 ^(*λ *= 1309 min) at *D *= 1 to 4.8 × 10^-2 ^min-^1 ^at *D *= 20 (*λ *= 131 min) (Fig. 5A). Thus the EGFR system with *D *= 20 generates accurate steady-state responses for input wavelengths greater than 131 min. Also, we have previously shown that the EGFR system with *D *= 20 responds with virtually no transient for *λ *greater than 96 min (Fig. 4). In all of our calculations, the critical wavelengths defined based on the steady-state bandwidth and the transient characteristics were close to each other with the former being slightly larger than the latter. Hence, below the bandwidth frequency the receptor systems considered here are capable of transducing the input pulse in an accurate fashion with the response rapidly settling onto a steady-state oscillation that tracks the input waveform. The GPCR system (Fig. 5B) displays qualitatively similar results. In this case, *ω*_*BW *_increases from 3 × 10^-5 ^s^-1 ^(*λ *= 2.1 × 10^5 ^s) at *k*_*ds *_= 6.5 × 10^-4 ^s^-1 ^to 1.7 × 10^-2 ^s^-1 ^(*λ *= 370 s) at *k*_*ds *_= 6.5 s^-1 ^(Fig. 5B). Overall, the increased response speed achieved by the downregulation and desensitization processes enables the receptor systems to operate in a larger input frequency range. This in turn helps to improve the temporal resolution of ligand sensing.

The steady-state time delay between the input and the output can be computed from the phase lag *ϕ *and it is an indicator of how well the cellular system can synchronize its output to the input stimuli. In the bottom-most panels of Figs. 5A and 5B we present the time-delay as a function of the input frequency for various magnitudes of receptor downregulation/desensitization. As seen in Fig. 5A for the EGFR system, when the input frequency is in the pass through region of the low-pass filter, the asymptotic time delay has a strong dependence on the extent of downregulation *D*. It drops from 211 min to 22 min when *D *is increased from 1 to 20. Clearly, receptor downregulation leads to improvements in input-output synchronization. Similar results are seen when the extent of GPCR desensitization is increased (Fig. 5B). For the GPCR the asymptotic delay drops from 32820 s at *k*_*ds *_= 6.5 × 10^-4 ^s^-1 ^to 62 s at *k*_*ds *_= 6.5 s^-1 ^which reflects significantly increased synchrony when desensitization rates are higher.

Our impulse response results for the EGFR system (Fig. 2) reveal that for downregulation values *D *> 6, the relaxation time of the response is less than 50 min. Furthermore, the frequency response results (Figs. 3, 4, 5) for *D *> 6 show that the EGFR system is capable of accurately processing inputs with wavelength greater than ~200 min. Experimental data from *in vitro *cell culture systems indicate that when a ligand bolus (impulse) is added to cells expressing EGFR, after a transient increase the receptor phosphorylation levels decay in a time-span of approximately 1 hour [33]. This suggests that the inherent time scale of the EGFR system is in line with the results obtained here using mathematical analysis of the system. We believe that the analysis methodology described here can serve as a tool to obtain indirect estimates of the time scales that the EGFR and GPCR systems are designed to process both under cell culture conditions and *in vivo*.

*Based on our observations, we conclude that the negative regulation of signaling systems via receptor downregulation and desensitization leads to speedy and synchronous processing of temporal ligand information*.

### Receptor downregulation/desensitization enhances the stability of the system resting state

**Figure 6:**
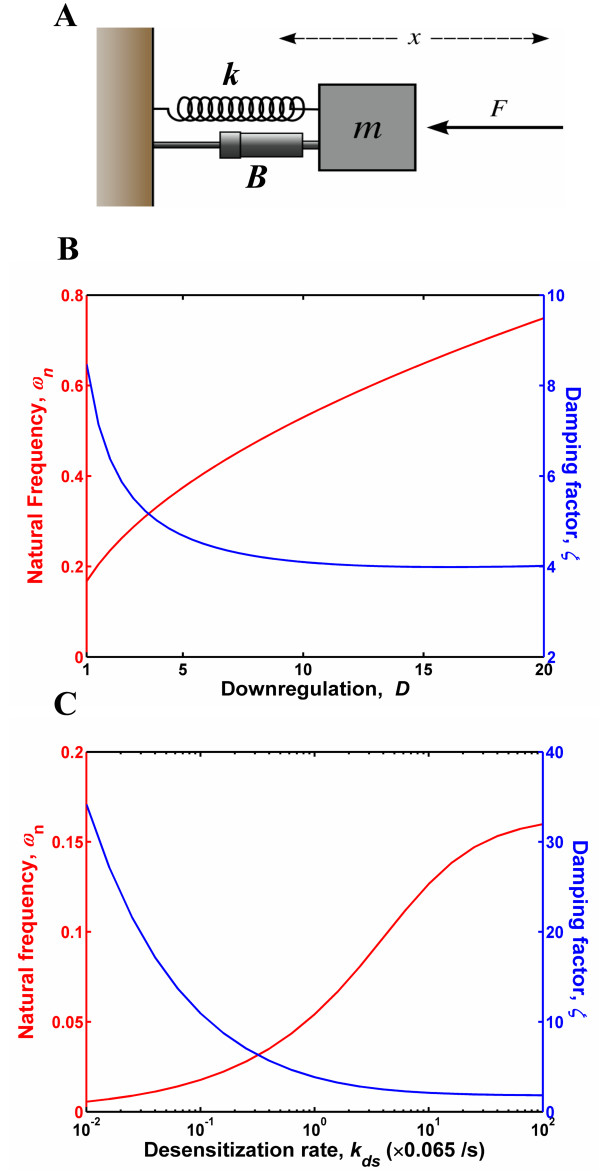
**EGFR and GPCR systems behave like over-damped mechanical oscillators**. (A) Schematic of a mass-spring-damper system. The system comprises of a mass connected to a wall via a spring with spring constant *k *(= *mω*_*n*_^2^) and a damper with damping constant *B *(= 2*mζω*_*n*_). The mass *m *is set in motion in response to the external force *F*, and the displacement *x *is a function of *ω*_*n *_the natural frequency and *ζ *the damping ratio. (B&C) Dependence of *ω*_*n *_(red line) and *ζ *(blue line) of the EGFR system on *D *(B) and of the GPCR system on *k*_*ds *_(C).

We analyzed the governing equations in order to identify the physical reasons behind the improvement in signal processing elicited by downregulation and desensitization. The linearized EGFR model is a second-order system (Eq. 1 in Methods). The behaviour of such second-order systems can be analyzed in the context of a classical harmonic oscillator. On the other hand, the linearized GPCR model is a fourth-order system (Eq. 2 in Methods), which can be conceptualized as two harmonic oscillators in series. However, to simplify the system description we identified an equivalent second-order system that would mimic the input-output behaviour of the GPCR transfer function. Figure 6A is a schematic of the classical second-order mass-spring-damper system that can be used as a physical reference frame for understanding our reaction networks. The input to the mechanical system is the external force *F*, which is analogous to the ligand entry rate *f*(*t*). The output of the mechanical system is the displacement *x*(*t*) of the attached mass *m*, which is in turn analogous to the output of the reaction systems (*C*(*t*) for the EGFR and *G*_*a*_(*t*) for the GPCR). The dynamics of the mass-spring-damper system can be characterized using two fundamental quantities: the natural frequency *ω*_*n *_and damping ratio *ζ*, which are in turn related to the force constant *k *of the spring and the damping constant *B *of the damper (or dashpot). The natural frequency quantifies the resilience of the system and it is related to the spring constant as ωn=k/m
 MathType@MTEF@5@5@+=feaafiart1ev1aaatCvAUfKttLearuWrP9MDH5MBPbIqV92AaeXatLxBI9gBaebbnrfifHhDYfgasaacPC6xNi=xH8viVGI8Gi=hEeeu0xXdbba9frFj0xb9qqpG0dXdb9aspeI8k8fiI+fsY=rqGqVepae9pg0db9vqaiVgFr0xfr=xfr=xc9adbaqaaeGacaGaaiaabeqaaeqabiWaaaGcbaacciGae8xYdC3aaSbaaSqaaiabd6gaUbqabaGccqGH9aqpdaGcaaqaamaalyaabaGaem4AaSgabaGaemyBa0gaaaWcbeaaaaa@333B@. The damping ratio *ζ *is given by ζ=B/(2mωn)=B/(2mk)
 MathType@MTEF@5@5@+=feaafiart1ev1aaatCvAUfKttLearuWrP9MDH5MBPbIqV92AaeXatLxBI9gBaebbnrfifHhDYfgasaacPC6xNi=xH8viVGI8Gi=hEeeu0xXdbba9frFj0xb9qqpG0dXdb9aspeI8k8fiI+fsY=rqGqVepae9pg0db9vqaiVgFr0xfr=xfr=xc9adbaqaaeGacaGaaiaabeqaaeqabiWaaaGcbaacciGae8NTdONaeyypa0JaemOqaiKaei4la8IaeiikaGIaeGOmaiJaemyBa0Mae8xYdC3aaSbaaSqaaiabd6gaUbqabaGccqGGPaqkcqGH9aqpcqWGcbGqcqGGVaWlcqGGOaakcqaIYaGmdaGcaaqaaiabd2gaTjabdUgaRbWcbeaakiabcMcaPaaa@407E@. Systems with higher natural frequencies correspond to stiffer springs and they rebound better when exposed to an external force. The damper on the other hand retards the free motion of the spring. A higher damping ratio corresponds to a more sluggish response to an external force.

Comparison of the governing equations for our model with those describing the motion of the mass in Fig. 6A enabled us to compute the natural frequency *ω*_*n *_and damping ratio *ζ *for the EGFR and GPCR systems. The effect of increasing the extent of receptor downregulation on the frequency and damping ratio of the EGFR system are shown in Fig. 6B. Increasing receptor downregulation both i) increases the frequency (red line), *i.e*., makes the system more resilient and ii) decreases the damping ratio (blue line), *i.e*., reduces the impedance on the restoring force provided by the spring. The latter effect can be understood as being analogous to the lubrication of the mechanical system. Increasing the desensitization rate in the GPCR model (Fig. 6C) has a qualitatively similar effect on the natural frequency and the damping ratio of this system.

*Overall, in the context of the mechanical analogy downregulation and desensitization can be metaphorically understood as phenomena that increase the "static stability" of receptor systems. They render the systems more "resilient" and thereby enable the response to bounce back in a rapid fashion following an external perturbation*.

## Conclusion

There is considerable evidence to suggest that evolution has optimized the regulation of the biomolecular networks so that they are efficient and function in a robust fashion [1,34-37]. The negative regulation of signaling systems via receptor internalization and desensitization is seen in a wide variety of signaling receptors [9,21,38,39]. It has been traditionally rationalized that these mechanisms are present in order to enable the signaling system to adapt to constant ligand stimulation [18,40]. However, under physiological conditions ligand stimulation can be expected to have spatio-temporal variations. Therefore, signaling systems are delegated with the task of decoding temporal ligand information. We used mathematical modelling to demonstrate that receptor downregulation and desensitization confer the EGFR and GPCR systems with an improved ability to process time-varying ligand concentrations. Thus, in addition to serving as a mechanism for signaling termination, downregulation and desensitization may play a key role in the context of information processing.

Establishing equivalencies between biological reaction networks and human engineered systems enabled us to uncover the functional role of the receptor downregulation/desensitization. We note that the molecular network underlying bacterial chemotaxis has been previously shown to employ a low-pass filter to ensure optimal noise filtering [41]. Analysis of the governing equations for receptor signaling systems studied here revealed that these systems are also similar to electronic low-pass filters but possibly with a different functional role.

In engineering systems, a low-pass filter provides the beneficial role of noise rejection and the system generates outputs where noise frequencies are suppressed. Thus, attenuation of frequencies above the bandwidth is viewed as *a desirable trait*. The biological receptor systems investigated here also suppress oscillations for frequencies beyond the bandwidth, but generate a non-zero steady-state response (*y*~*y*_*mean*_) to high frequency inputs. Let us consider the case of a sinusoidal input with frequency higher than the bandwidth. This input would traditionally be viewed as "noise" in the context of engineering but it would still elicit a biological response that can be viewed here as an inaccurate reconstruction of the input – i.e., a constant steady-state response. We suggest that in these biological systems frequencies beyond the bandwidth are not necessarily noise; they may contain useful temporal information that the system is simply incapable of processing. In other words, being a low-pass filter can be viewed as an *undesirable constraint that restricts *the range of input frequencies that the receptor systems can accurately process. Receptor downregulation and desensitization benefit the system by increasing the bandwidth of the low-pass filter, thus enabling the system to process this previously inaccessible range of input frequencies. This finding could be particularly relevant in developmental biology where a cell is presented with the task of making critical decisions based on temporally varying ligand information. Receptor downregulation and desensitization would enable the cell in a developing embryo to perceive ligand information using a finer temporal resolution, and to generate appropriate biological responses that are an accurate translation of the environmental cues that it receives.

In addition to the analogy with electrical circuitry, we established the parallelism between the cellular reaction networks and a classical mechanical over-damped mass-spring-damper system. In the context of this analogy downregulation and desensitization can be metaphorically understood as phenomena that increase the "static stability" of receptor systems. They render the systems more "resilient" and thereby enable the response to bounce back in a rapid fashion following an external perturbation.

We believe that such characterizations of biomolecular networks in the context of engineering or physical systems will significantly improve our understanding of cellular networks. The mathematical analysis methodology detailed in this paper is relatively simple and we employ techniques that are well-established in control systems literature. We believe that the novelty of our work lies in our demonstration that even simple engineering-based analyses can reveal a lot about the underlying design principles of biomolecular networks. Our analysis helped us uncover non-intuitive rationales for the existence of negative regulation mechanisms in receptor signaling networks. Following the elucidation of proteomes and protein interaction maps, an increasing class of biological problems deals with answering the questions of what a specific reaction network does and how it achieves its functionality [4,42]. Addressing these questions will enable us to move towards identifying how a network can be modified either to rectify faulty behaviour or to generate desired responses. In this regard, engineering metaphors such as the ones described here could prove to be invaluable in overcoming the barriers that biological complexity imposes on our ability to understand these systems.

## Methods

### Governing equations and numerical solution

Ordinary differential equations (ODEs) for the EGFR and GPCR models depicted in Fig. 1 were formulated in the standard way assuming mass action kinetics for the various reaction steps. Detailed descriptions of the model equations and their solution procedures are provided in the Additional file 1. A brief overview of our analysis methodology follows.

Numerical solutions of the EGFR and GPCR ODE systems were obtained by integrating the ODEs (Additional file 1) using the ODE15s stiff equation solver of MATLAB (Mathworks, Natick, MA). For all simulations the rate constants used were those listed in Table S1 in the Additional file 1 unless specified otherwise.

### Analytical solution of the EGFR and GPCR models

Investigation of the parameter dependencies in the models becomes easier when analytical solutions are available. The non-linearity of the governing equations for the EGFR and GPCR models prevents us from obtaining exact analytical solutions for these systems. However, the response of the models to relatively small perturbations can be reasonably approximated by solving the system of equations obtained by linearization of the model around the initial steady-state. We solved linearized versions of the EGFR and GPCR governing equations using the technique of Laplace transforms (Additional file 1). Briefly, taking the Laplace transform of the linear ODE systems yields algebraic equations from which the relevant transfer functions can be extracted. The transfer functions completely describe the input-output relationships in the underlying model. For the EGFR system the dimensionless transfer function can be written as:

G(s)=1s2+(1+β+γ)s+βγ
 MathType@MTEF@5@5@+=feaafiart1ev1aaatCvAUfKttLearuWrP9MDH5MBPbIqV92AaeXatLxBI9gBaebbnrfifHhDYfgasaacPC6xNi=xI8qiVKYPFjYdHaVhbbf9v8qqaqFr0xc9vqFj0dXdbba91qpepeI8k8fiI+fsY=rqGqVepae9pg0db9vqaiVgFr0xfr=xfr=xc9adbaqaaeGacaGaaiaabeqaaeqabiWaaaGcbaGaem4raCKaeiikaGIaem4CamNaeiykaKIaeyypa0tcfa4aaSaaaeaacqaIXaqmaeaacqWGZbWCdaahaaqabeaacqaIYaGmaaGaey4kaSIaeiikaGIaeGymaeJaey4kaSccciGae8NSdiMaey4kaSIae83SdCMaeiykaKIaem4CamNaey4kaSIae8NSdiMae83SdCgaaaaa@4391@

Here *β *= *k*_*e*_/*k*_*off *_and *γ *= *k*_*on*_*R*_*T*_/(*k*_*off*_*N*_*av*_*V*) are dimensionless system parameters. *V *is the extracellular volume per cell and *N*_*av *_is Avogadro's number. *β *and *γ *respectively represent the ability of the system to capture extracellular ligand molecules and the ability of a receptor system to consume bound ligand [29]. The EGFR model is thus characterized by a second-order transfer function that relates the input f*(t*) to the number of surface receptor-ligand complexes C*(t*). The input-output characteristics of the GPCR model are captured by a fourth-order transfer function that relates the input f*(t*) to the number of active G-protein molecules *G*_a_*(t*). This transfer function can be written as:

G(s)=φfρf(s+φr)[(s+γ)(s+η)ρf+s(1+s+γ)(s+η+ρr)]
 MathType@MTEF@5@5@+=feaafiart1ev1aaatCvAUfKttLearuWrP9MDH5MBPbIqV92AaeXatLxBI9gBaebbnrfifHhDYfgasaacPC6xNi=xI8qiVKYPFjYdHaVhbbf9v8qqaqFr0xc9vqFj0dXdbba91qpepeI8k8fiI+fsY=rqGqVepae9pg0db9vqaiVgFr0xfr=xfr=xc9adbaqaaeGacaGaaiaabeqaaeqabiWaaaGcbaGaem4raCKaeiikaGIaem4CamNaeiykaKIaeyypa0tcfa4aaSaaaeaaiiGacqWFgpGzdaWgaaqaaiabdAgaMbqabaGae8xWdi3aaSbaaeaacqWGMbGzaeqaaaqaaiabcIcaOiabdohaZjabgUcaRiab=z8aMnaaBaaabaGaemOCaihabeaacqGGPaqkdaWadaqaamaabmaabaGaem4CamNaey4kaSIae83SdCgacaGLOaGaayzkaaWaaeWaaeaacqWGZbWCcqGHRaWkcqWF3oaAaiaawIcacaGLPaaacqWFbpGCdaWgaaqaaiabdAgaMbqabaGaey4kaSIaem4CamNaeiikaGIaeGymaeJaey4kaSIaem4CamNaey4kaSIae83SdCMaeiykaKYaaeWaaeaacqWGZbWCcqGHRaWkcqWF3oaAcqGHRaWkcqWFbpGCdaWgaaqaaGqaciab+jhaYbqabaaacaGLOaGaayzkaaaacaGLBbGaayzxaaaaaaaa@6343@

The six dimensionless system parameters in Eq. 2 are: *ϕ*_*f *_= *k*_*a*_*R*_*T*_/*k*_*off*_, *ϕ*_*r *_= *k*_*i*_/*k*_*off*_, *ρ*_*f *_= *k*_*fr*_/*k*_*off*_, *ρ*_*r *_= *k*_*rr*_/*k*_*off*_, *γ *= *k*_*on*_*R*_*T*_/(*k*_*off*_*N*_*av*_*V*), and *η *= *k*_*ds*_*/k*_*off*_.

#### Impulse response

We used the transfer functions to analyze the response of the receptor models to time-varying ligand inputs. The system output to any input f*(t*) can be obtained by taking the inverse Laplace transform of the product *G*(*s*)*f*(*s*) where *f*(*s*) is the Laplace transform of the input. For the case of a ligand impulse of magnitude 0.01*K*_*D *_(dimensionless magnitude = 0.01), *f*(*s*) is simply the constant 0.01. Hence we computed the impulse response by inverting the transfer function and multiplying the result by the constant 0.01. For the range of parameter values employed in our analysis, the poles of both the EGFR and GPCR transfer functions are always real, distinct and negative. Hence both systems are stable and their dynamic response can be obtained as a sum of exponentials as y(t∗)=0.01∑i=1,nKiepit∗
 MathType@MTEF@5@5@+=feaafiart1ev1aaatCvAUfKttLearuWrP9MDH5MBPbIqV92AaeXatLxBI9gBaebbnrfifHhDYfgasaacPC6xNi=xH8viVGI8Gi=hEeeu0xXdbba9frFj0xb9qqpG0dXdb9aspeI8k8fiI+fsY=rqGqVepae9pg0db9vqaiVgFr0xfr=xfr=xc9adbaqaaeGacaGaaiaabeqaaeqabiWaaaGcbaGaemyEaKNaeiikaGIaemiDaq3aaWbaaSqabeaacqGHxiIkaaGccqGGPaqkcqGH9aqpcqaIWaamcqGGUaGlcqaIWaamcqaIXaqmdaaeqbqaaiabdUealnaaBaaaleaacqWGPbqAaeqaaOGaemyzau2aaWbaaSqabeaacqWGWbaCdaWgaaadbaGaemyAaKgabeaaliabdsha0naaCaaameqabaGaey4fIOcaaaaaaSqaaiabdMgaPjabg2da9iabigdaXiabcYcaSiabd6gaUbqab0GaeyyeIuoaaaa@47C0@ where *y*(t*) corresponds to the dimensionless system output (C* for EGFR and *G*_a_* for GPCR), *p*_*i *_are the roots of the characteristic polynomial in the denominator of Eqs. 1 and 2, *n *is the order of the polynomial (*n *= 2 for EGFR and *n *= 4 for GPCR) and *K*_*i *_are coefficients obtained during partial-fractions expansion of the transfer functions (see Additional file 1). Once the impulse response was computed as above, we quantified the speed of the impulse response using the relaxation time. The relaxation time is defined as the time taken for the response to decay to 1/*e *of its maximum. We interpolated the analytical response curve to determine the relaxation time.

#### Frequency response analysis

In order to determine the range of input frequencies the receptor systems can process, we computed the response of the linearized EGFR and GPCR models to sinusoidal inputs of the form *f*(*t*) = (*A*/2) [1 - cos(*ωt*)]. Hence, the ligand entry rate starts from a value of 0 and varies sinusoidally between 0 and a peak value of *A *nM/min with a frequency *ω *radians/min. In dimensionless terms, the input can be written as f*(t*) = (A*/2) [1 - cos(ω* t*)] where A* = *A*/(*K*_*D*_*k*_*off*_), ω* = *ω*/*k*_*off*_, t* = *tk*_*off *_and *k*_*off *_and *K*_*D *_are respectively the dissociation rate and the dissociation constant for the receptor-ligand binding reaction. The Laplace transform of the input is given by *f*(*s*) = (A*/2)ω*2/[*s*(*s*^2 ^+ ω*2)]. Again, the response of the EGFR and GPCR models to this sinusoidal input can be obtained by finding the inverse Laplace transform of the product *G*(*s*)*f*(*s*), where *G*(*s*) is the corresponding transfer function. The frequency response can be written as *y*(t*) = *y*_*tr*_(t*) + *y*_*ss*_(t*) where *y*_*tr*_(t*) and *y*_*ss*_(t*) are the transient and steady-state contributions to the response, respectively. The transient term can be expressed as ytr(t∗)=(A∗ω∗2/2)∑i=1,nTi(ω∗)epit∗
 MathType@MTEF@5@5@+=feaafiart1ev1aaatCvAUfKttLearuWrP9MDH5MBPbIqV92AaeXatLxBI9gBaebbnrfifHhDYfgasaacPC6xNi=xH8viVGI8Gi=hEeeu0xXdbba9frFj0xb9qqpG0dXdb9aspeI8k8fiI+fsY=rqGqVepae9pg0db9vqaiVgFr0xfr=xfr=xc9adbaqaaeGacaGaaiaabeqaaeqabiWaaaGcbaGaemyEaK3aaSbaaSqaaiabdsha0jabdkhaYbqabaGccqGGOaakcqWG0baDdaahaaWcbeqaaiabgEHiQaaakiabcMcaPiabg2da9iabcIcaOiabdgeabnaaCaaaleqabaGaey4fIOcaaGGacOGae8xYdC3aaWbaaSqabeaacqGHxiIkcqaIYaGmaaGccqGGVaWlcqaIYaGmcqGGPaqkdaaeqbqaaiabdsfaunaaBaaaleaacqWGPbqAaeqaaOGaeiikaGIae8xYdC3aaWbaaSqabeaacqGHxiIkaaGccqGGPaqkcqWGLbqzdaahaaWcbeqaaiabdchaWnaaBaaameaacqWGPbqAaeqaaSGaemiDaq3aaWbaaWqabeaacqGHxiIkaaaaaaWcbaGaemyAaKMaeyypa0JaeGymaeJaeiilaWIaemOBa4gabeqdcqGHris5aaaa@557D@ where *T*_*i*_(ω*) are frequency-dependent coefficients (provided in Additional file 1) obtained in taking the inverse Laplace transform of the product *G*(*s*)*f*(s) and the other terms have been defined previously. Since the *p*_*i *_values for the EGFR and GPCR transfer functions are real and negative, the transient term- like the name suggests- exponentially reaches a value of zero. Further, we can show that *y*_*tr *_*rises *from a negative value at t* = 0 to a value of zero (Additional file 1). The steady-state term in the frequency response can be expressed as *y*_ss_(t*) = |*y*_mean _- (A*/2)|*G*(iω*)|cos(ω*t* + *ϕ*)| where *y*_*mean *_is the mean value of the steady-state response, and |*G*(iω*)| and *ϕ *are respectively the magnitude and phase of the complex number *G*(iω*). Here, *y*_*mean *_is a time-invariant constant that is a function of the poles of the transfer function. As shown in the Additional file 1, *y*_*mean *_= A*/(2*p*_1_*p*_2_) for the EGFR system and it is equal to – A*ρ_*f *_*ϕ*_*f*_/(2*p*_1_*p*_2_*p*_3_*ϕr*) for the GPCR system. Hence, the steady-state response is a sinusoidal oscillation about a constant mean value *y*_*mean *_with an amplitude ratio (output amplitude/input amplitude) given by |*G*(iω*)|. Further, *y*_*ss *_trails the sinusoidal input f*(t*) with a phase lag of *ϕ*. Overall, the response of the EGFR and GPCR systems to a non-negative sinusoidal variation in the ligand entry rate comprises a rise from zero to a steady-state mean value of *y*_*mean *_about which the response continues to oscillate. We characterized the frequency response of our receptor systems by examining the transient and steady-state terms individually.

In order to quantify the transient response, we computed the time *t*_99 _in minutes taken for the magnitude of *y*_*tr*_(t*) to decay by 99% (i.e., to 1% of its value at t* = 0) for a range of input frequencies and system parameters for the EGFR and GPCR systems. Since *y*_*tr*_(t*) rises from a negative number at t* = 0 to a value of zero, *t*_99 _characterizes the time taken for the response to rise to steady-state. We computed the dimensionless normalized rise time as *t*_99_/*λ*, and this quantity gives us the number of input cycles necessary before the output settles into the steady-state response.

As mentioned earlier, the steady-state frequency response of the receptor systems can be characterized by computing the frequency dependent response function *G*(iω*). The amplitude ratio *AR *of the response and the phase lag *ϕ *respectively are given by the magnitude and the phase of the complex number *G*(iω*). We computed the *AR *and *ϕ *values for the EGFR and GPCR models for a range of input frequencies at specific values of the respective system parameters. Once we obtained *AR *vs. *ω *curves, we computed the bandwidth frequency *ω*_*BW*_, which is defined as the frequency at which the output amplitude of the system drops to 70.7% of the zero frequency amplitude. We also computed the time-lag between the input and the output waveforms as *t*_*delay *_= *ϕ*/*ω *(in units of minutes).

### Analysis of the models in the context of classical second-order dynamics

As seen above, the EGFR response model is a second-order system. Second-order systems can be understood in the context of a classical mechanical oscillator. The transfer function of a canonical second-order system can be written as *G*(*s*) = *K*/(*s*^2 ^+ 2*ζω*_*n *_s + *ω*_*n*_^2^) where *K *is the gain, *ζ *is the damping ratio, and *ω*_*n *_is the natural frequency of the oscillator. Comparing this expression with the actual transfer function of the EGFR model, we find that *ω*_*n *_= βγ
 MathType@MTEF@5@5@+=feaafiart1ev1aaatCvAUfKttLearuWrP9MDH5MBPbIqV92AaeXatLxBI9gBaebbnrfifHhDYfgasaacPC6xNi=xH8viVGI8Gi=hEeeu0xXdbba9frFj0xb9qqpG0dXdb9aspeI8k8fiI+fsY=rqGqVepae9pg0db9vqaiVgFr0xfr=xfr=xc9adbaqaaeGacaGaaiaabeqaaeqabiWaaaGcbaWaaOaaaeaaiiGacqWFYoGycqWFZoWzaSqabaaaaa@2F38@ and *ζ *= (1 + *β *+ *γ*)/(2βγ
 MathType@MTEF@5@5@+=feaafiart1ev1aaatCvAUfKttLearuWrP9MDH5MBPbIqV92AaeXatLxBI9gBaebbnrfifHhDYfgasaacPC6xNi=xH8viVGI8Gi=hEeeu0xXdbba9frFj0xb9qqpG0dXdb9aspeI8k8fiI+fsY=rqGqVepae9pg0db9vqaiVgFr0xfr=xfr=xc9adbaqaaeGacaGaaiaabeqaaeqabiWaaaGcbaWaaOaaaeaaiiGacqWFYoGycqWFZoWzaSqabaaaaa@2F38@). For the EGFR system, we can show that *ζ *> 1 for all *β*, *γ *> 0. Hence, this system behaves as an over-damped second order system. We used these expressions to compute the natural frequency and the damping ratio values for the EGFR system.

The linearized GPCR model is a fourth-order system. Such systems can be conceptually described as two second-order systems (harmonic oscillators) connected in series. However, we sought to simplify the description of the GPCR system by finding a second-order transfer function that would approximate the behaviour of the fourth-order system. For a given parameter set we first computed the fourth-order transfer function *G*(*s*) using Eq. 2. Subsequently we computed a second-order transfer function Γ (*s*) that would display similar input-output characteristics as *G*(*s*) (Additional file 1). Once Γ (*s*) was determined, the coefficients of its quadratic denominator were compared to the characteristic polynomial of the canonical second-order transfer function to obtain the corresponding *ζ *and *ω*_*n *_values.

## Authors' contributions

HS, HSW and HR jointly designed the study and wrote the paper, and HS performed the research. All authors read and approved the final manuscript.

## Supplementary Material

Additional File 1 Supplementary Methods. Detailed descriptions of numerical and analytical solution methodology and rate constants employed in the mathematical model. Click here for file
