# Supplementary material for: Receptor downregulation and desensitization enhance the information processing ability of signalling receptors
Source: BMC Syst Biol. 2007 Nov 9;1:48. doi: 10.1186/1752-0509-1-48 (PMC2228318; doi:10.1186/1752-0509-1-48)
Supplement: Additional File 1 — Supplementary Methods. Detailed descriptions of numerical and analytical solution methodology and rate constants employed in the mathematical model. [file 1752-0509-1-48-S1.doc]

**SUPPLEMENTARY MATERIAL**

**Governing equations and numerical solution**

Dimensional and dimensionless versions of the governing equations for the EGFR and GPCR models are presented in Table S1, which also presents the rate constants employed in the simulations. For the EGFR model the binding kinetics (*k_on_* and *k_off_*) values are from [1] and the trafficking (*k_e_* and *k_t_*) values are from [2]. The receptor expression levels *R_T_* are for EGFR in human mammary epithelial cells and the volume *V* is in the range that is typically employed in *in vitro* experiments. When there is no extracellular ligand present *L*(*t*)*=C*(*t*)*=*0, we obtain the relationship *V_R_* = *k_t_R_T_* for the zero-order receptor synthesis rate. This expression states that the total number of surface receptors prior to ligand addition reflects the balance between receptor synthesis and internalization terms. In all we have six independent parameters in the model: *k_on_*, *k_off_*, *k_e_*, *k_t_*, *V*, and *R_T_*.

The GPCR model used here is a simplified version of the one presented in the work of Riccobene et al. [3]. These authors employ a cyclical reaction network to implement receptor activation. Their network consists of 4 species: inactive free receptors, *R*; active free receptors *R*^*^; inactive complexes (*LR* ≡ *C* in our model); and active complexes (*LR*^*^ ≡ *C_a_* in our model). There are four reversible reactions between these species, which in cyclical order are as follows: 1) receptor-ligand binding (*R*+*L* ↔ *LR*) with association constant *K_bind_*; 2) activation of receptor-ligand complexes (*LR* ↔ *LR^*^*) with equilibrium constant *αK_act_*; 3) ligand dissociation from active complexes (*LR^*^* ↔ *R^*^*+*L*) with dissociation constant 1/*αK_bind_*; and 4) deactivation of free receptors (*R^*^* ↔ *R*) with equilibrium deactivation constant 1/*K_act_*. Here *α* is the effect of ligand binding on receptor activation (Reaction 2 compared with 4) and due to the cyclical nature of the network also quantifies the effect of receptor activation on ligand dissociation (Reaction 1 compared with 3). We used *α*=10^4^, which is the suggested value in Riccobene et al. This results in only a small number of active free receptors *R^*^*, which motivated us to simplify our model by choosing a single linear route towards receptor activation viz. (R+L ↔ *C* ↔ *C_a_*). While, this simplification results in a delay in G-protein activation and a slight decrease in the overall amplitude of the response compared to the full cyclical model, the qualitative behavior of the model is preserved. Further, Riccobene et al. assume that desensitized receptors reversibly bind to extracellular ligand with an affinity much smaller than that of normal receptors. We have eliminated this infrequently occurring reaction in order to reduce the dimensionality of our model.

Numerical solutions of the EGFR and GPCR ordinary differential equation (ODE) systems were obtained by integrating the governing equations (Table S1) using the ODE15s stiff equation solver of the MATLAB program with the appropriate initial conditions. For the impulse response calculations, the initial conditions used were *R*=*R_T_*, *C=*0, and *L*=0.01*K_D_* for the EGFR model and *R*=*R_T_*, *G*=*G_T_*, *C*=*C_a_*=*C_d_*=*G_a_*=0, and *L*=0.01*K_D_* for the GPCR model. Here *K_D_*=*k_off_*/*k_on_* is the dissociation constant. Unless specified otherwise, rate constants listed in Table S1 were used in the simulations.

**Linearization and analytical solution of a non-linear ODE system**

Consider a system of non-linear ordinary-differential equations describing a general reaction network: d***x***/d*t* = ***M***(***x***). Here ***x*** is the vector of species concentrations, *t* is the time variable, and ***M*** is a vector of non-linear functions describing the rate of change of species concentrations. This non-linear system can be linearized around the initial condition ***x***=***x***_0_ by using Taylor series expansions for the non-linear functions *M_i_* around the initial steady-state. Defining a set of deviation variables ***x’*=*x*−*x*_0_**, and discarding the higher order terms in the Taylor series expansion of ***M***, we obtain d***x’***/d*t* = (∂***M***(***x’***)**/**∂***x’*)|*_x’_*_=0_*x’***, where (∂***M***(***x’***)**/**∂***x’*)|*_x’_*_=0_** is the Jacobian matrix for the system evaluated at ***x*’=0.** At the initial steady state ***M***(***x*’=0**)**=**0 so there is no constant term in the expansion. Hence, we can write the linear ODE system as d***x’***/d*t* = ***Jx’*** with ***J*** representing the Jacobian at ***x’*=0**. The *J_ij_* element of the ***J*** matrix is the derivative of the function *M_i_* with respect to the variable *x_j_*. If a set of external disturbances are imposed on this system, then the governing equations become d***x’***/d*t* = ***Jx’* + *F***(*t*) where ***F***(*t*) is a vector of disturbances with *F_i_*(*t*) describing the time-dependent disturbance in the *i*^th^ species. This linearized equation system is presented in Table S1 for the EGFR and GPCR models.

The linear system of equations obtained as described above can be solved using Laplace transforms with the initial conditions ***x’***=0. Taking Laplace transforms of the linear ODE system, results in the algebraic system (*s****I*−*J*) *x’***(*s*) **= *F***(*s*) where ***I*** is an identity matrix with the same dimensions as ***J***, and ***x’***(*s*) and ***F***(*s*) are the Laplace transforms of ***x’***(*t*) and ***F***(*t*), respectively. The solution of this system can be written as ***x’*(*s*) = *G***(*s*)***F***(*s*) where ***G***(*s*) **=** (*s****I*−*J*)^−^**^1^ is the transfer function matrix that relates the external system inputs ***F*** to the outputs ***x’***. We used this relationship to determine the transfer function matrix for the EGFR and GPCR models. The element *G_ij_* of the transfer function matrix relates the *j^th^* input to the concentration of the *i*^th^ species. In our models, the dimensionless ligand production rate *f^*^*(*t^*^*) is the only externally imposed disturbance. Hence the transfer functions of interest for the EGFR and GPCR models are the *G*_32_ and *G*_72_ elements of their respective ***G***(*s*) matrices. These transfer function elements are shown in Table S1 (and in Eqs. 1 and 2 of the manuscript) for the two models. As seen, the EGFR response is characterized by a second-order transfer function while the GPCR response is fourth-order.

**Impulse response of the linearized EGFR and GPCR models**

We inverted the transfer functions using the standard partial-fractions technique to obtain the dynamic response. Figure S1 reports how the poles of the EGFR and GPCR transfer functions depend on the downregulation and desensitization parameters. The poles of both the EGFR and GPCR models were found to be real, negative and distinct over the range of parameter values employed in the manuscript. Due to the nature of these systems we plot the magnitude of the real poles as a function of the parameters rather than presenting the results in an *imaginary axis* vs. *real axis* root-locus plot as done traditionally (e.g., see [4, 5]). The results indicate that both systems are stable and the solutions can be expressed as a linear combination of exponential decays with the number of exponential terms being equal to the order of the characteristic polynomial. For a ligand impulse of dimensionless magnitude 0.01 the response of the EGFR and GPCR models can be written in a general form as *y*(*t^*^*) =where *y*(*t^*^*) corresponds to the dimensionless system output (*C^*^* for EGFR and *G_a_^*^* for GPCR), *n* is the order of the system (*n*=2 for EGFR and *n*=4 for GPCR), *p_i_* are the roots of the characteristic polynomial in the denominator of the transfer functions, and *K_i_* are coefficients obtained during partial-fractions expansion of the transfer functions. The analytical solution for the impulse response of the second-order EGFR model can be expressed as:

(S1)

In the above equation *p*_1,2_ are the roots of the quadratic characteristic polynomial for the EGFR system: *s*^2^ *+* (*1+β+γ*)*s +βγ* . The fourth-order transfer function for the GPCR model when inverted yields the following equation for the response to an impulse of magnitude 0.01:

(S2)

We define the three roots of the cubic polynomial in decreasing order of magnitude with |*p*_1_|>|*p*_2_|>|*p*_3_| in the discussions.

**Response of the linearized EGFR and GPCR models to sinusoidal inputs**

In order to examine the frequency response of the EGFR and GPCR systems we chose a non-negative sinusoidal function for the ligand entry rate, *f^*^*(*t^*^*) = (*A^*^*/2)[1−cos(*ω^*^t^*^*)]. The dimensionless response *y*(*t^*^*) to this input can be obtained by taking the inverse Laplace transform of the product *G*(*s*)*f*(*s*) where *f*(*s*)= (*A^*^*/2) *ω^*^*2/[*s*(*s*^2^+*ω^*^*^2^)]. For the EGFR system the frequency response can be written as:

(S3)

In Eq. S3, |*G*(*iω^*^*)| and *φ* respectively are the magnitude and phase of the complex number *G*(*iω^*^*). Since the poles of the EGFR system, *p*_1_ and *p*_2_ are real and negative, the magnitude of the first term in curly braces in Eq. S3 will decay to zero. For |*p*_1_| > |*p*_2_|, the second exponential term will be negative and will be larger than the first exponential term, thereby resulting in an overall negative value. Thus, the term in the first curly braces of Eq. S3 is a transient rise from a negative value to 0 and is denoted as *y_tr_*(*t*^*^) in the manuscript. The second term in curly braces in Eq. S3 is the steady-state response *y_ss_*(*t*^*^). This term constitutes a sinusoidal variation about a mean value of *A*^*^/(2*p*_1_*p*_2_). Further the steady-state response has an amplitude ratio of |*G*(*iω^*^*)| and trails the input with a phase lag of *φ* radians. The frequency response of the GPCR system can be derived in a similar fashion and can be expressed as:

(S4)

Similar to the EGFR frequency response, Eq. S4 for the GPCR response is the sum of a transient (term in the first curly braces) and a steady-state response (term in the second curly braces).

**Second-order approximation for the fourth-order GPCR transfer function**

The GPCR model is characterized by a fourth-order transfer function with roots *p*_1_, *p*_2_, *p*_3_ and −*φ_r_*. The roots *p*_1_, *p*_2_, *p*_3_ are real and negative. Of the four roots *p*_1_ has the largest magnitude and was found to be approximately one to two orders-of-magnitude larger than the other three (Fig. S1). Hence this root was discarded as first step towards identifying a lower order approximation for *G*(s). The third-order transfer function approximation was obtained as below taking care to match the gains of the two transfer functions. We first write the transfer function *G*(*s*) in its fully factored form as:

(S3)

Now, since *p*_1_ is the largest root we get:

(S4)

Note that since *p*_1_ is negative *G*_3_(s) still possesses a positive gain. This third-order transfer-function was further approximated by an equivalent second-order system using the method of Hsia [6]. Briefly, we first expressed the transfer function *G*_3_(s) in the form *G*_3_(*s*) = *K*/(*l*_3_s^3^ + *l*_2_s^2^ + *l*_1_s + 1). We then derived coefficients *f*_2_ and *f*_4_ from the coefficients of the cubic polynomial using the equations: *f*_2_ = 2*l*_2_ – *l*_1_^2^ and *f*_4_ = *l*_2_^2^ – 2*l*_1_*l*_3_. The unknown coefficients of the second-order approximating transfer function *Γ*(*s*) = *G*_2_(s) = *K*/(*d*_2_s^2^ + *d*_1_s + 1) were then computed using the relations: *d*_2_ = $\sqrt{f_{4}}$ and *d*_1_ = $\sqrt{2d_{2}-f_{2}}$. The impulse response of the full fourth-order transfer function *G*(*s*) and its second-order approximation *Γ*(*s*) are compared in Fig. S2 for the parameter values listed in Table S1. As seen the second-order transfer function serves as an adequate approximation for the GPCR model. Although *ζ* and *ω_n_* can be analytically described in terms of the dimensionless GPCR parameters, these equations are unwieldy and are not presented here.

**REFERENCES**

1. Hendriks BS, Orr G, Wells A, Wiley HS, Lauffenburger DA: **Parsing ERK activation reveals quantitatively equivalent contributions from epidermal growth factor receptor and HER2 in human mammary epithelial cells.** *J Biol Chem* 2005, **280:**6157-6169.

2. Resat H, Ewald JA, Dixon DA, Wiley HS: **An integrated model of epidermal growth factor receptor trafficking and signal transduction.** *Biophys J* 2003, **85:**730-743.

3. Riccobene TA, Omann GM, Linderman JJ: **Modeling activation and desensitization of G-protein coupled receptors provides insight into ligand efficacy.** *J Theor Biol* 1999, **200:**207-222.

4. Chaudhary N, Bhartiya S, Venkatesh KV: **System-level analysis of tryptophan regulation in Escherichia coli--performance under starved and well-fed conditions.** *IET Syst Biol* 2007, **1:**181-189.

5. Yang L, Iglesias PA: **Positive feedback may cause the biphasic response observed in the chemoattractant-induced response of Dictyostelium cells.** *Syst Control Lett* 2006, **55:**329-337.

6. Hsia TC: **On the simplification of linear systems.** *IEEE Trans Automatic Control* 1972, **AC-17:**372-374.

**Table 1:** Governing equations and parameter values for the EGFR and GPCR models

|  | **EGFR** | | | **GPCR** | | |
| --- | --- | --- | --- | --- | --- | --- |
| Governing equations | Dimensional  d*R*/*d t*= –*k_on_RL* + *k_off_C* – *k_t_R* + *V_R_*  d*L*/d*t* = (–*k_on_ RL* + *k_off_C*)/(*N_av_V*) + *f*(*t*)  d*C*/d*t = k_on_RL* – *k_off_C* – *k_e_C* | | Dimensionless*^a^*  d*R^*^*/*dt^*^*= –*R^*^L^*^* + *C^*^* –*α (R^*^* – 1)  d*L^*^*/d*t^*^*= *γ* [– *R^*^L^*^* + *C^*^*] + *f^*^*(*t^*^*)  d*C^*^*/d*t^*^= R^*^L^*^* – (1+*β)C^*^* | Dimensional  d*R*/d*t* = –*k_on_RL* + *k_off_C*  d*L*/d*t* = (–*k_on_ RL* + *k_off_C*)/(*N_av_V*) + *f*(*t*)  d*C*/d*t* = *k_on_ RL* – *k_off_C* − *k_fr_C* + *k_rr_C_a_*  d*C_a_*/d*t* = *k_fr_C* − *k_rr_C_a_* − *k_ds_C_a_*  d*C_d_*/d*t* = *k_ds_ C_a_*  d*G*/d*t* = −*k_a_GC_a_* + *k_i_G_a_*  d*G_a_*/d*t* = *k_a_GC_a_* − *k_i_G_a_* | | Dimensionless*^a^*  d*R^*^*/d*t^*^* = −*R^*^L^*^* + *C^*^*  d*L^*^*/d*t^*^* = *γ* [−*R^*^L^*^* + *C^*^*] + *f^*^*(*t^*^*)  d*C^*^*/d*t^*^* = *R^*^L^*^* − *C^*^* − *ρ_f_C^*^* + *ρ_r_C_a_^*^*  d*C_a_^*^*/d*t^*^* = *ρ_f_C^*^* − *ρ_r_C_a_^*^* − *η C_a_^*^*  d*C_d_^*^*/d*t^*^* = *η C_a_^*^*  d*G^*^*/d*t^*^* = −*φ_f_G^*^C_a_^*^* + *φ_r_G_a_^*^*  d*G_a_^*^*/d*t^*^* = *φ_f_G^*^C_a_^*^* − *φ_r_G_a_^*^* |
| Linearized equations:  d***x'****/dt=* ***J***_0_⋅***x’*** +***F*(*t*)** |  | | |  | | |
| Transfer function^b^ |  | | |  | | |
| Parameter values^c^ | *k_on_* ­= 0.097 /nM/min |  | | *k_on_* = 8.4×10^7^ /M/s |  | |
|  | *k*_off_ = 0.24 /min | Dimensionless parameters: | | *k_off_* = 0.37 /s | Dimensionless parameters: | |
|  | *k_e_*  = 0.15 /min | *α* = *k_t_*/*k_off_* = 0.083 | | *k_fr_* = 10 /s | *γ = K_a_R­_T_*/(*N_av_V*) = 0.052 | |
|  | *k_t_* = 0.02 /min | *β* = *k_e_*/*k_off_* = 0.63 | | *k_rr_* = 10 /s | *ρ_f_ = k_fr_*/*k_off_* = 27.03 | |
|  | *R_T_* = 2×10^5^ | *γ* = *K_a_R­_T_*/(*N_av_V*) = 0.34 | | *k_ds_* = 0.065 /s | *ρ_r_ = k_rr_*/*k_off_* = 27.03 | |
|  | *V* = 4×10^−10^ liters/cell |  | | *k_a_* = 10^−7^ /s | *η = k_ds_/k_off_* = 0.176 | |
|  |  |  | | *k_i_* = 2×10^−1^ /s | *φ_f_ = k_a_R_T_/k_off_* = 0.015 | |
|  |  |  | | *R_T_* = 5.5×10^4^ | *φ_r_* = *k_i_*/*k_off_* = 0.541 | |
|  |  |  | | *G_T_* = 1×10^5^ |  | |
|  |  |  | | *V* = 4×10^−10^ liters/cell |  | |

*^a^* The dimensionless variables are defined as follows: **EGFR** – *t^*^* = *k_off_t; R^*^* = *R*/*R_T_*; *C^*^* = *C*/*R_T_*; *L^*^* = *L*/*K_D_*; **GPCR** - *t^*^* = *k_off_t; R^*^* = *R*/*R_T_*; *C^*^* = *C*/*R_T_*; *L^*^* = *L*/*K_D_*; *C_a_^*^* = *C_a_*/*R_T_*; *C_d_^*^* = *C_d_*/*R_T_* ; *G^*^* = *G*/*G_T_*; *G_a_^*^* = *G_a_*/*G_T_  ^b^*The transfer function relates the ligand input *f*(*t*) *C^*^* for the EGFR model and *G_a_^*^* for the GPCR model ^c^ **EGFR:** *k_off_* and *K_D_* are from [1] and the rest from [2]. **GPCR:** All rate constants are from [3]. The volume, *V* is in the range typically seen in *in vitro* experiments.


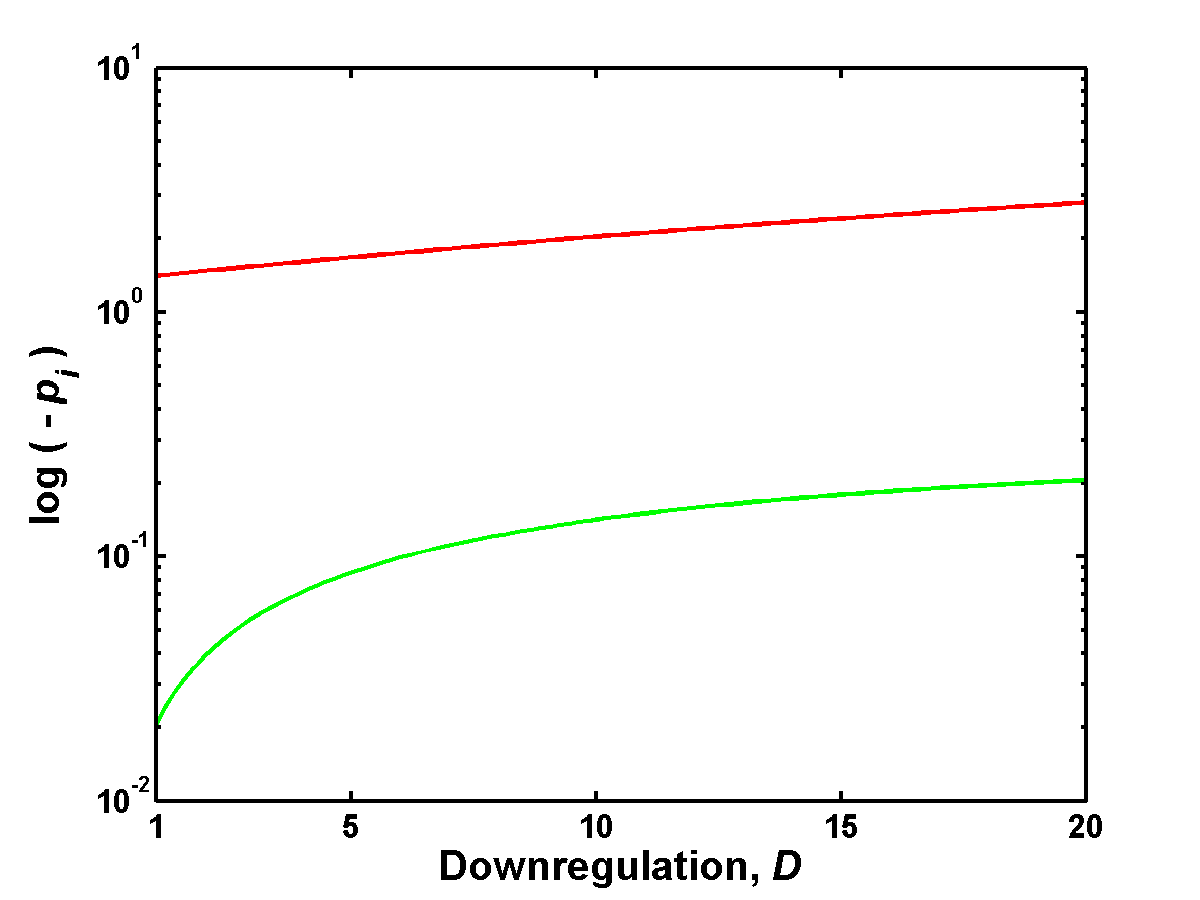

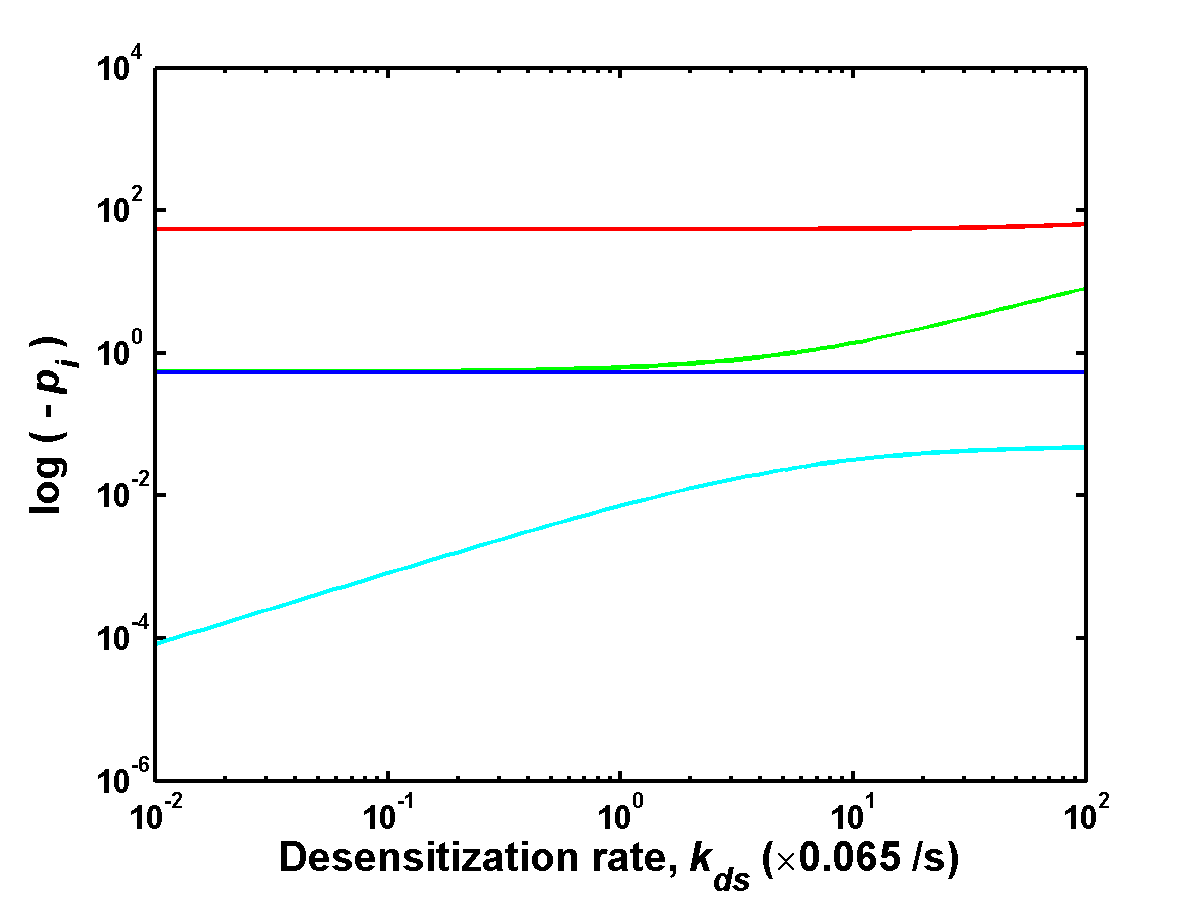


**Figure S1: Poles of the EGFR and GPCR transfer functions.** The absolute values of the poles of the transfer functions are plotted on a log-scale as a function of the extent of downregulation, *D* for the EGFR (A) and the desensitization rate, *k_ds_* for the GPCR (B) systems. For the EGFR system the two poles vary from −1.4 to −2.8 (red) and −0.02 to −0.21 (green) when *D* is varied from 1 to 20. For the GPCR system, three of the poles vary from −54.6 to −65.6 (red), −0.55 to −8.0 (green) and −1×10^−4^ to −4.7×10^−2^ (cyan) respectively, while the fourth pole stays constant at −0.541 (= −*φ*_r_) (blue) when the desensitization rate is varied. Both systems have real, negative and distinct poles for the entire range of parameter values simulated. Further, the trends in the poles of the systems suggest that the systems would respond faster when downregulation/desensitization is increased.

**B**

**A**

**Figure S2: Second-order approximation for the fourth-order GPCR transfer function.** The response of the GPCR system to a ligand impulse of dimensionless magnitude 0.01 is plotted for a range of desensitization rates. The second-order transfer function (dotted lines) yields results that are in reasonable agreement with the response of the actual fourth-order transfer function (solid lines).


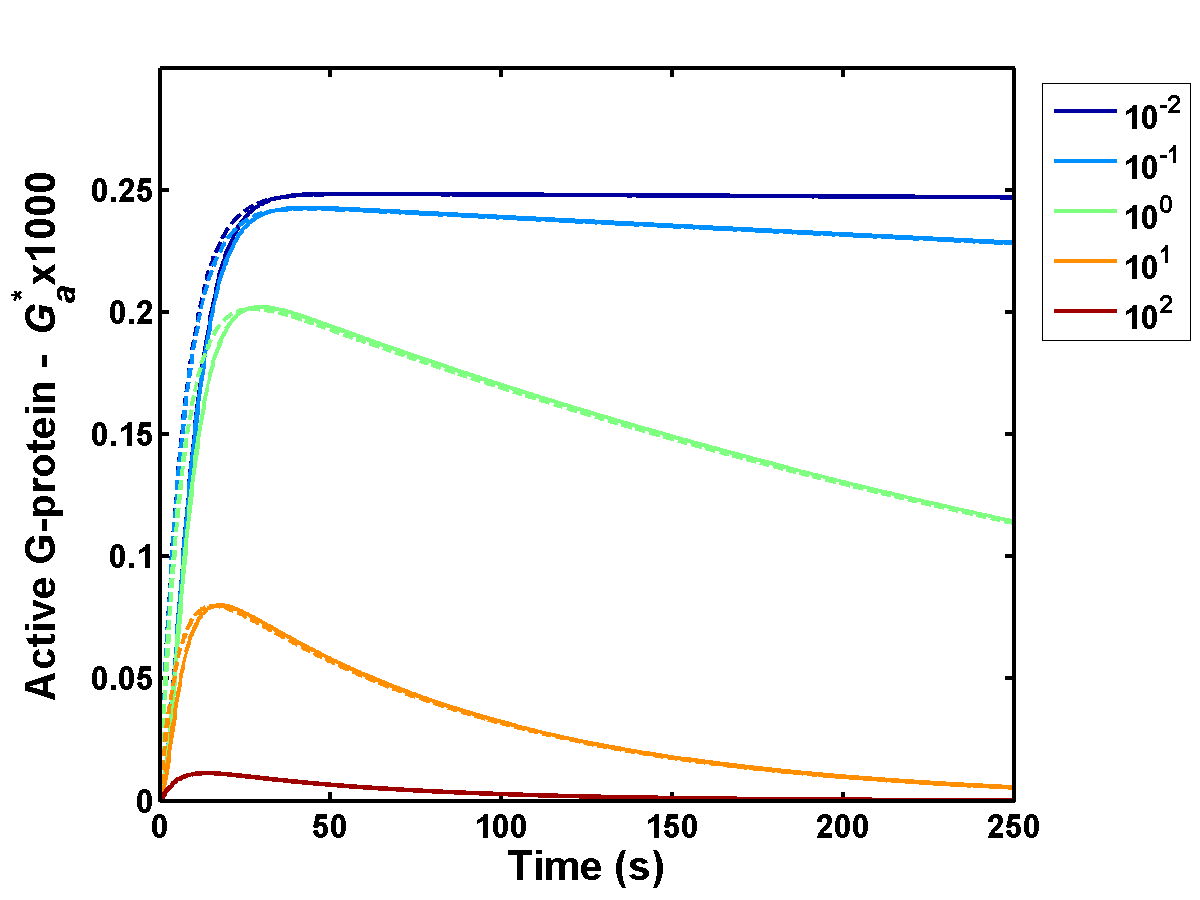


***k_ds_* (x0.065 /s)**
